# *tert*-Butyl *N*′-[4-(2-pyrid­yl)benzyl­idene]hydrazinecarboxyl­ate

**DOI:** 10.1107/S1600536809012835

**Published:** 2009-04-25

**Authors:** Qi Feng, Qiong Tang, Hao Xu, Cheng Yao

**Affiliations:** a Department of Applied Chemistry, College of Science, Nanjing University of Technology, Nanjing 210009, People’s Republic of China

## Abstract

In the mol­ecule of the title compound, C_17_H_19_N_3_O_2_, the aromatic rings are oriented at a dihedral angle of 3.68 (3)°. In the crystal structure, inter­molecular N—H⋯O hydrogen bonds link the mol­ecules into chains along the *a* axis. A weak C—H⋯π inter­action is also present.

## Related literature

For a related structure, see: Sugi *et al.* (2002 ▶). For bond-length data, see: Allen *et al.* (1987 ▶).
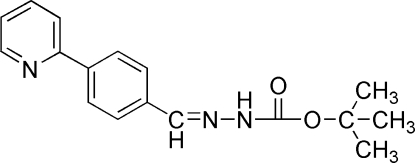


## Experimental

### 

#### Crystal data

C_17_H_19_N_3_O_2_
*M* *_r_* = 297.35
Monoclinic, 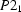

*a* = 5.3080 (11) Å
*b* = 6.3010 (13) Å
*c* = 23.459 (5) Å
β = 91.01 (3)°
*V* = 784.5 (3) Å^3^
*Z* = 2
Mo *K*α radiation
μ = 0.08 mm^−1^
*T* = 294 K
0.30 × 0.20 × 0.10 mm

#### Data collection

Enraf–Nonius–Nonius CAD-4 diffractometer
Absorption correction: ψ scan (North *et al.*, 1968 ▶) *T*_min_ = 0.975, *T*_max_ = 0.992
1743 measured reflections
1566 independent reflections
1249 reflections with *I* > 2σ(*I*)
*R*_int_ = 0.028
3 standard reflections frequency: 120 min intensity decay: 1%

#### Refinement

*R*[*F* ^2^ > 2σ(*F* ^2^)] = 0.051
*wR*(*F* ^2^) = 0.170
*S* = 1.00
1566 reflections
199 parameters
1 restraint
H-atom parameters constrained
Δρ_max_ = 0.16 e Å^−3^
Δρ_min_ = −0.21 e Å^−3^

### 

Data collection: *CAD-4 Software* (Enraf–Nonius, 1985 ▶); cell refinement: *CAD-4 Software*; data reduction: *XCAD4* (Harms & Wocadlo, 1995 ▶); program(s) used to solve structure: *SHELXS97* (Sheldrick, 2008 ▶); program(s) used to refine structure: *SHELXL97* (Sheldrick, 2008 ▶); molecular graphics: *PLATON* (Spek, 2009 ▶); software used to prepare material for publication: *SHELXTL* (Sheldrick, 2008 ▶).

## Supplementary Material

Crystal structure: contains datablocks I, global. DOI: 10.1107/S1600536809012835/hk2661sup1.cif

Structure factors: contains datablocks I. DOI: 10.1107/S1600536809012835/hk2661Isup2.hkl

Additional supplementary materials:  crystallographic information; 3D view; checkCIF report

## Figures and Tables

**Table 1:** Hydrogen-bond geometry (Å, °)

*D*—H⋯*A*	*D*—H	H⋯*A*	*D*⋯*A*	*D*—H⋯*A*
N1—H1*A*⋯O2^i^	0.86	2.30	3.113 (5)	158
C16—H16*A*⋯*Cg*2^ii^	0.93	2.80	3.588 (4)	144

Symmetry codes: (i) 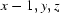; (ii) 
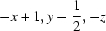
. *Cg*2 is the centroid of the N3/C13–C17 ring.

**Fig. 1.**
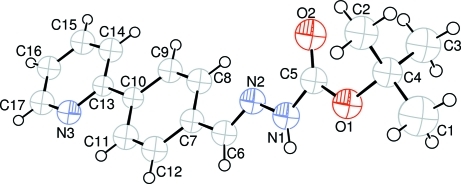
The molecular structure of the title molecule, with the atom-numbering scheme.

**Fig. 2.**
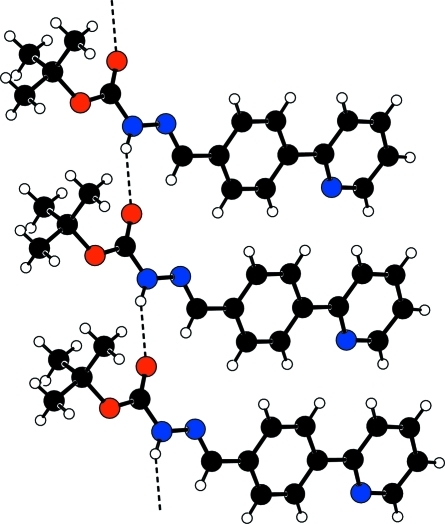
A partial packing diagram of the title compound. Hydrogen bonds are shown as dashed lines.

## supplementary crystallographic information

### Comment

The title compound is an important intermediate in the syntheses of medicines. We report herein its crystal structure.

In the molecule of the title compound (Fig 1), the bond lengths (Allen *et al.*,  1987) and angles are within normal ranges. Rings A (C7-C12) and B (N3/C13-C17) are, of course, planar, and they are oriented at a dihedral angle of 3.68 (3)°. So, they are nearly coplanar.

In the crystal structure, intermolecular N-H···O hydrogen bonds (Table 1) link the molecules into chains along the a axis, in which they may be effective in the stabilization of the structure. There also exists a weak C—H···π interaction (Table 1).

### Experimental

The title compound was prepared according to a literature method (Sugi *et al.*,  2002). Crystals suitable for X-ray analysis were obtained by dissolving the title compound (1.5 g) in methanol (25 ml) and evaporating the solvent slowly at room temperature for about 5 d.

### Refinement

H atoms were positioned geometrically, with N-H = 0.86 Å (for NH) and C-H = 0.93 and 0.96 Å for aromatic and methyl H, respectively, and constrained to ride on their parent atoms, with U_iso_(H) = xU_eq_(C,N), where x = 1.5 for methyl H and x = 1.2 for all other H atoms. The absolute structure could not be determined reliably, and 338 Friedel pairs were averaged before the last cycle of refinement.

### Crystal data

**Table:**

C_17_H_19_N_3_O_2_	*F*(000) = 316
*M**_r_* = 297.35	*D*_x_ = 1.259 Mg m^−^^3^
Monoclinic, *P*2_1_	Mo *K*α radiation, λ = 0.71073 Å
Hall symbol: P 2yb	Cell parameters from 25 reflections
*a* = 5.3080 (11) Å	θ = 9–13°
*b* = 6.3010 (13) Å	µ = 0.08 mm^−^^1^
*c* = 23.459 (5) Å	*T* = 294 K
β = 91.01 (3)°	Needle, colorless
*V* = 784.5 (3) Å^3^	0.30 × 0.20 × 0.10 mm
*Z* = 2	

### Data collection

**Table:**

Nonius–Nonius CAD-4 diffractometer	1249 reflections with *I* > 2σ(*I*)
Radiation source: fine-focus sealed tube	*R*_int_ = 0.028
graphite	θ_max_ = 25.3°, θ_min_ = 1.7°
ω/2θ scans	*h* = 0→6
Absorption correction: ψ scan (North *et al.*, 1968)	*k* = 0→7
*T*_min_ = 0.975, *T*_max_ = 0.992	*l* = −28→28
1743 measured reflections	3 standard reflections every 120 min
1566 independent reflections	intensity decay: 1%

### Refinement

**Table:**

Refinement on *F*^2^	Primary atom site location: structure-invariant direct methods
Least-squares matrix: full	Secondary atom site location: difference Fourier map
*R*[*F*^2^ > 2σ(*F*^2^)] = 0.051	Hydrogen site location: inferred from neighbouring sites
*wR*(*F*^2^) = 0.170	H-atom parameters constrained
*S* = 1.00	*w* = 1/[σ^2^(*F*_o_^2^) + (0.1*P*)^2^ + 0.33*P*] where *P* = (*F*_o_^2^ + 2*F*_c_^2^)/3
1566 reflections	(Δ/σ)_max_ < 0.001
199 parameters	Δρ_max_ = 0.16 e Å^−^^3^
1 restraint	Δρ_min_ = −0.21 e Å^−^^3^

### Special details

**Table:**

Geometry. All e.s.d.'s (except the e.s.d. in the dihedral angle between two l.s. planes) are estimated using the full covariance matrix. The cell e.s.d.'s are taken into account individually in the estimation of e.s.d.'s in distances, angles and torsion angles; correlations between e.s.d.'s in cell parameters are only used when they are defined by crystal symmetry. An approximate (isotropic) treatment of cell e.s.d.'s is used for estimating e.s.d.'s involving l.s. planes.
Refinement. Refinement of *F*^2^ against ALL reflections. The weighted *R*-factor *wR* and goodness of fit *S* are based on *F*^2^, conventional *R*-factors *R* are based on *F*, with *F* set to zero for negative *F*^2^. The threshold expression of *F*^2^ > σ(*F*^2^) is used only for calculating *R*-factors(gt) *etc*. and is not relevant to the choice of reflections for refinement. *R*-factors based on *F*^2^ are statistically about twice as large as those based on *F*, and *R*- factors based on ALL data will be even larger.

### Fractional atomic coordinates and isotropic or equivalent isotropic displacement parameters (Å^2^)

**Table:**

	*x*	*y*	*z*	*U*_iso_*/*U*_eq_	
O1	0.5696 (5)	1.5114 (6)	0.37089 (14)	0.0589 (9)	
O2	0.8570 (6)	1.3033 (6)	0.32757 (16)	0.0688 (10)	
N1	0.4371 (6)	1.2553 (6)	0.31524 (16)	0.0524 (9)	
H1A	0.2891	1.3001	0.3235	0.063*	
N2	0.4651 (7)	1.0839 (6)	0.27985 (16)	0.0507 (9)	
N3	0.1499 (6)	0.2707 (7)	0.06762 (16)	0.0545 (10)	
C1	0.5928 (11)	1.7923 (13)	0.4341 (3)	0.099 (2)	
H1B	0.5025	1.8791	0.4071	0.149*	
H1C	0.6959	1.8808	0.4583	0.149*	
H1D	0.4751	1.7155	0.4569	0.149*	
C2	0.9263 (10)	1.7590 (10)	0.3637 (2)	0.0684 (14)	
H2B	0.8254	1.8437	0.3381	0.103*	
H2C	1.0257	1.6612	0.3423	0.103*	
H2D	1.0356	1.8496	0.3859	0.103*	
C3	0.9018 (11)	1.4977 (13)	0.4441 (2)	0.0815 (18)	
H3A	1.0048	1.4006	0.4233	0.122*	
H3B	0.7854	1.4195	0.4669	0.122*	
H3C	1.0069	1.5841	0.4684	0.122*	
C4	0.7575 (8)	1.6377 (9)	0.40286 (19)	0.0569 (12)	
C5	0.6416 (8)	1.3524 (7)	0.3368 (2)	0.0506 (10)	
C6	0.2709 (8)	1.0167 (8)	0.25407 (19)	0.0505 (10)	
H6A	0.1184	1.0872	0.2581	0.061*	
C7	0.2841 (8)	0.8282 (8)	0.21781 (17)	0.0476 (10)	
C8	0.4830 (8)	0.6875 (9)	0.2220 (2)	0.0544 (12)	
H8A	0.6092	0.7110	0.2493	0.065*	
C9	0.4994 (8)	0.5144 (8)	0.18695 (19)	0.0532 (11)	
H9A	0.6369	0.4236	0.1905	0.064*	
C10	0.3124 (7)	0.4726 (7)	0.14596 (16)	0.0410 (9)	
C11	0.1129 (8)	0.6120 (8)	0.1429 (2)	0.0567 (12)	
H11A	−0.0145	0.5891	0.1159	0.068*	
C12	0.0966 (8)	0.7830 (9)	0.17841 (19)	0.0566 (12)	
H12A	−0.0441	0.8706	0.1759	0.068*	
C13	0.3321 (7)	0.2892 (7)	0.10648 (16)	0.0412 (9)	
C14	0.5270 (9)	0.1443 (8)	0.1101 (2)	0.0564 (12)	
H14A	0.6522	0.1580	0.1381	0.068*	
C15	0.5332 (9)	−0.0208 (9)	0.0717 (2)	0.0612 (13)	
H15A	0.6635	−0.1194	0.0735	0.073*	
C16	0.3498 (9)	−0.0394 (8)	0.0314 (2)	0.0566 (12)	
H16A	0.3513	−0.1501	0.0052	0.068*	
C17	0.1603 (9)	0.1099 (9)	0.0301 (2)	0.0590 (12)	
H17A	0.0346	0.0991	0.0021	0.071*	

### Atomic displacement parameters (Å^2^)

**Table:**

	*U*^11^	*U*^22^	*U*^33^	*U*^12^	*U*^13^	*U*^23^
O1	0.0443 (15)	0.052 (2)	0.081 (2)	−0.0021 (15)	0.0055 (14)	−0.0195 (18)
O2	0.0480 (17)	0.057 (2)	0.102 (2)	0.0030 (18)	0.0129 (16)	−0.022 (2)
N1	0.0446 (18)	0.042 (2)	0.070 (2)	0.0005 (18)	0.0065 (15)	−0.015 (2)
N2	0.057 (2)	0.036 (2)	0.059 (2)	0.0007 (18)	0.0094 (16)	−0.0084 (18)
N3	0.0498 (19)	0.047 (2)	0.066 (2)	−0.001 (2)	−0.0026 (16)	−0.010 (2)
C1	0.070 (3)	0.100 (6)	0.129 (5)	−0.010 (4)	0.012 (3)	−0.068 (5)
C2	0.072 (3)	0.055 (3)	0.078 (3)	−0.008 (3)	−0.005 (2)	0.007 (3)
C3	0.081 (3)	0.096 (5)	0.066 (3)	−0.007 (4)	−0.020 (2)	0.018 (4)
C4	0.050 (2)	0.055 (3)	0.065 (3)	−0.004 (2)	0.003 (2)	−0.011 (3)
C5	0.049 (2)	0.035 (2)	0.067 (3)	0.003 (2)	0.0086 (19)	−0.008 (2)
C6	0.046 (2)	0.044 (2)	0.061 (2)	−0.001 (2)	0.0100 (18)	−0.006 (2)
C7	0.048 (2)	0.044 (2)	0.051 (2)	−0.003 (2)	0.0120 (17)	−0.001 (2)
C8	0.052 (2)	0.049 (3)	0.062 (3)	0.010 (2)	−0.0083 (19)	−0.011 (2)
C9	0.049 (2)	0.042 (2)	0.068 (3)	0.010 (2)	−0.0021 (19)	−0.004 (2)
C10	0.0413 (19)	0.041 (2)	0.0405 (18)	−0.0016 (19)	0.0030 (14)	0.0036 (18)
C11	0.046 (2)	0.050 (3)	0.074 (3)	0.003 (2)	−0.008 (2)	−0.010 (3)
C12	0.042 (2)	0.056 (3)	0.072 (3)	0.014 (2)	−0.0091 (19)	−0.010 (3)
C13	0.0380 (18)	0.040 (2)	0.0461 (19)	−0.004 (2)	0.0050 (15)	−0.0028 (19)
C14	0.059 (3)	0.046 (3)	0.064 (3)	0.009 (2)	0.003 (2)	−0.001 (2)
C15	0.061 (3)	0.049 (3)	0.073 (3)	0.011 (3)	0.009 (2)	−0.003 (3)
C16	0.062 (3)	0.046 (3)	0.062 (3)	−0.004 (2)	0.008 (2)	−0.013 (2)
C17	0.061 (3)	0.055 (3)	0.061 (3)	−0.008 (3)	−0.002 (2)	−0.013 (3)

### Geometric parameters (Å, °)

**Table:**

O1—C5	1.341 (5)	C6—C7	1.463 (6)
O1—C4	1.471 (5)	C6—H6A	0.9300
O2—C5	1.208 (5)	C7—C12	1.376 (6)
N1—C5	1.338 (6)	C7—C8	1.381 (6)
N1—N2	1.372 (5)	C8—C9	1.369 (7)
N1—H1A	0.8600	C8—H8A	0.9300
N2—C6	1.259 (6)	C9—C10	1.395 (6)
N3—C13	1.322 (5)	C9—H9A	0.9300
N3—C17	1.344 (6)	C10—C11	1.377 (6)
C1—C4	1.508 (8)	C10—C13	1.486 (6)
C1—H1B	0.9600	C11—C12	1.365 (7)
C1—H1C	0.9600	C11—H11A	0.9300
C1—H1D	0.9600	C12—H12A	0.9300
C2—C4	1.503 (7)	C13—C14	1.381 (6)
C2—H2B	0.9600	C14—C15	1.377 (7)
C2—H2C	0.9600	C14—H14A	0.9300
C2—H2D	0.9600	C15—C16	1.351 (7)
C3—C4	1.508 (8)	C15—H15A	0.9300
C3—H3A	0.9600	C16—C17	1.377 (7)
C3—H3B	0.9600	C16—H16A	0.9300
C3—H3C	0.9600	C17—H17A	0.9300
			
C5—O1—C4	120.6 (3)	C7—C6—H6A	119.8
C5—N1—N2	119.6 (3)	C12—C7—C8	117.2 (4)
C5—N1—H1A	120.2	C12—C7—C6	121.2 (4)
N2—N1—H1A	120.2	C8—C7—C6	121.6 (4)
C6—N2—N1	117.3 (4)	C9—C8—C7	121.7 (4)
C13—N3—C17	118.7 (4)	C9—C8—H8A	119.1
C4—C1—H1B	109.5	C7—C8—H8A	119.1
C4—C1—H1C	109.5	C8—C9—C10	120.8 (4)
H1B—C1—H1C	109.5	C8—C9—H9A	119.6
C4—C1—H1D	109.5	C10—C9—H9A	119.6
H1B—C1—H1D	109.5	C11—C10—C9	116.9 (4)
H1C—C1—H1D	109.5	C11—C10—C13	121.8 (4)
C4—C2—H2B	109.5	C9—C10—C13	121.3 (4)
C4—C2—H2C	109.5	C12—C11—C10	121.9 (4)
H2B—C2—H2C	109.5	C12—C11—H11A	119.0
C4—C2—H2D	109.5	C10—C11—H11A	119.0
H2B—C2—H2D	109.5	C11—C12—C7	121.4 (4)
H2C—C2—H2D	109.5	C11—C12—H12A	119.3
C4—C3—H3A	109.5	C7—C12—H12A	119.3
C4—C3—H3B	109.5	N3—C13—C14	121.5 (4)
H3A—C3—H3B	109.5	N3—C13—C10	116.1 (4)
C4—C3—H3C	109.5	C14—C13—C10	122.5 (3)
H3A—C3—H3C	109.5	C15—C14—C13	119.1 (4)
H3B—C3—H3C	109.5	C15—C14—H14A	120.5
O1—C4—C2	111.7 (4)	C13—C14—H14A	120.5
O1—C4—C3	110.1 (5)	C16—C15—C14	119.9 (5)
C2—C4—C3	112.8 (4)	C16—C15—H15A	120.1
O1—C4—C1	101.7 (4)	C14—C15—H15A	120.1
C2—C4—C1	109.0 (5)	C15—C16—C17	118.3 (5)
C3—C4—C1	111.0 (5)	C15—C16—H16A	120.9
O2—C5—N1	125.4 (4)	C17—C16—H16A	120.9
O2—C5—O1	125.4 (4)	N3—C17—C16	122.6 (4)
N1—C5—O1	109.2 (3)	N3—C17—H17A	118.7
N2—C6—C7	120.3 (4)	C16—C17—H17A	118.7
N2—C6—H6A	119.8		
			
C5—N1—N2—C6	−170.0 (5)	C13—C10—C11—C12	−178.8 (4)
C5—O1—C4—C2	63.9 (6)	C10—C11—C12—C7	2.3 (7)
C5—O1—C4—C3	−62.3 (5)	C8—C7—C12—C11	−3.3 (7)
C5—O1—C4—C1	−180.0 (5)	C6—C7—C12—C11	177.3 (4)
N2—N1—C5—O2	0.7 (7)	C17—N3—C13—C14	−1.8 (6)
N2—N1—C5—O1	−178.7 (3)	C17—N3—C13—C10	178.8 (4)
C4—O1—C5—O2	−2.8 (7)	C11—C10—C13—N3	1.4 (6)
C4—O1—C5—N1	176.6 (4)	C9—C10—C13—N3	−176.9 (4)
N1—N2—C6—C7	−177.0 (4)	C11—C10—C13—C14	−178.0 (4)
N2—C6—C7—C12	−163.2 (5)	C9—C10—C13—C14	3.7 (6)
N2—C6—C7—C8	17.4 (7)	N3—C13—C14—C15	1.1 (7)
C12—C7—C8—C9	2.5 (7)	C10—C13—C14—C15	−179.6 (4)
C6—C7—C8—C9	−178.1 (4)	C13—C14—C15—C16	−0.2 (7)
C7—C8—C9—C10	−0.8 (7)	C14—C15—C16—C17	0.1 (7)
C8—C9—C10—C11	−0.3 (7)	C13—N3—C17—C16	1.7 (7)
C8—C9—C10—C13	178.0 (4)	C15—C16—C17—N3	−0.8 (7)
C9—C10—C11—C12	−0.4 (7)		

### Hydrogen-bond geometry (Å, °)

**Table:**

*D*—H···*A*	*D*—H	H···*A*	*D*···*A*	*D*—H···*A*
N1—H1A···O2^i^	0.86	2.30	3.113 (5)	158
C16—H16A···Cg2^ii^	0.93	2.80	3.588 (4)	144

Symmetry codes: (i) *x*−1, *y*, *z*; (ii) −*x*+1, *y*−1/2, −*z*.
